# Oxidative Stress in Endurance Flight: An Unconsidered Factor in Bird Migration

**DOI:** 10.1371/journal.pone.0097650

**Published:** 2014-05-15

**Authors:** Susanne Jenni-Eiermann, Lukas Jenni, Shona Smith, David Costantini

**Affiliations:** 1 Swiss Ornithological Institute, Sempach, Switzerland; 2 Institute for Biodiversity, Animal Health and Comparative Medicine, University of Glasgow, Glasgow, United Kingdom; 3 Department of Biology, University of Antwerp, Wilrijk, Belgium; Arizona State University, United States of America

## Abstract

Migrating birds perform extraordinary endurance flights, up to 200 h non-stop, at a very high metabolic rate and while fasting. Such an intense and prolonged physical activity is normally associated with an increased production of reactive oxygen and nitrogen species (RONS) and thus increased risk of oxidative stress. However, up to now it was unknown whether endurance flight evokes oxidative stress. We measured a marker of oxidative damage (protein carbonyls, PCs) and a marker of enzymatic antioxidant capacity (glutathione peroxidase, GPx) in the European robin (*Erithacus rubecula*), a nocturnal migrant, on its way to the non-breeding grounds. Both markers were significantly higher in European robins caught out of their nocturnal flight than in conspecifics caught during the day while resting. Independently of time of day, both markers showed higher concentrations in individuals with reduced flight muscles. Adults had higher GPx concentrations than first-year birds on their first migration. These results show for the first time that free-flying migrants experience oxidative stress during endurance flight and up-regulate one component of antioxidant capacity. We discuss that avoiding oxidative stress may be an overlooked factor shaping bird migration strategies, e.g. by disfavouring long non-stop flights and an extensive catabolism of the flight muscles.

## Introduction

Endurance flight of migratory birds is an extraordinary physical performance carried out at a very high metabolic rate which is about twice the maximum rate of exercising small mammals [1]–[2]. Moreover, this high metabolic rate is maintained during non-stop flights of up to 200 h in certain species and while fasting [3]. With the exception of aerial feeders, migrants do not feed during endurance flight and, thus, have to rely exclusively on body stores for energy and water. Endurance flight of birds is fuelled with energy derived from protein (at least 5% in fat birds) and lipids (at most 95%) [1] In comparison, mammals (including humans) rely to a much smaller proportion on lipids during strenuous endurance exercise, e.g. 40–50% in marathon runners [4]. Endurance flight of birds is performed at 60–85% maximal oxygen uptake (VO_2max_), or more when birds are loaded with fuel [5].

Several particular physiological adaptations have been found which apparently enable migratory birds to perform such an extraordinary endurance exercise. They include special mechanisms of lipid supply from adipose tissue to the mitochondria of the flight muscles, and a very high oxidative capacity of the flight muscles to catabolize lipids (reviewed in [2]). Also there is no indication that muscle fatigue or the lack of sleep limits the duration of non-stop flights in birds [2], [6]. It thus appears that migratory birds have evolved ways to overcome limitations applying to mammals regarding overall metabolic rate, lipid catabolism, protein sparing and duration of endurance exercise.

Intense and prolonged physical activity is normally associated with an increased production of reactive oxygen and nitrogen species (RONS) and thus the risk of oxidative stress, as observed in several mammalian species and humans [7]–[9]. During exercise the generation of free radicals occurs predominantly in skeletal and heart muscle and in blood [8]–[11]. If the production of RONS is not adequately balanced by antioxidants, oxidative damage to biomolecules occurs [12].

However, the oxidative balance has not yet been investigated in migrating birds. On the one hand, the very high metabolic rate and extremely long flight durations would speak in favour of a considerable increase in RONS production. Moreover physical activity may cause inflammation and activation of immune cells, which can increase oxidative stress further [7]. On the other hand, an increase in metabolic activity is not accompanied by a proportional increase in free radical production [13]. Muscle fatigue, a typical consequence of oxidative stress, is obviously not observed in birds [3]. Therefore, migrating birds may well show adaptations to set bounds to RONS production and up-regulate antioxidant capacity accordingly.

Studies available up to now investigating oxidative balance in birds during the migratory phase or during prolonged flight were restricted to homing pigeons which are efficient flyers, but not real migrants, and to migrating passerines during rest at a stop-over site. In homing pigeons, the redox status was shifted towards more oxidative conditions (oxidative damage increased while serum antioxidant capacity decreased) after a 5.2 h flight than after a 1.3 h or no flight [14]. In two passerine species resting after a migratory flight, there was some evidence that plasma non-enzymatic antioxidants were higher, and the balance between oxidative damage and antioxidants in plasma better, in birds with higher body energy stores [15].

In this study we investigated a marker of oxidative damage and a marker of enzymatic antioxidant capacity in red blood cells of a migrant passerine, the European robin, caught out of migratory flight at a Swiss Alpine Pass. There, migrants can be caught out of their nocturnal migratory flight towards the non-breeding grounds. We chose the European robin because it is the only nocturnal migrant which uses the Alpine pass for resting and feeding at day in noticeable numbers. Thus we could compare for the first time in a migratory bird markers of oxidative stress in two metabolic phases, the phase of extremely high metabolic rate during endurance exercise while fasting and the phase of low metabolic rate during resting and foraging. We show that oxidative stress is occurring during endurance flight and may be a factor shaping bird migration.

## Materials and Methods

### Ethic statement

Capture and blood-sampling on Col de Bretolet (see below) was done under licenses of the Office Vétérinaire du Canton du Valais (No. VS 15.1) and the Federal Office for the Environment in Bern (ringer license F044–0799), Switzerland. After blood sampling all birds were ringed and released immediately. All methods were approved by the ethics commission of the Office Vétérinaire du Canton du Valais.

### Animals and study site

Free-living European robins were caught in mist nets on Col de Bretolet (1923 asl), an Alpine pass located at the border between Switzerland and France (46°09′N; 06°47′E; for details, see [16]) during their autumn migratory period (September–October) in 2011 (N = 49) and 2012 (N = 52). European robins are nocturnal migrants. At night, individuals were caught exclusively in high mist nets of 8.5 m height put across the crest of the pass. Thus they were considered to be in active migratory flight. At dawn, robins land in large numbers to rest throughout the day. During the day, individuals were caught only in mist nets of about 2 m height placed in bushes and were considered resting and foraging. They may fly short distances while searching for an appropriate resting site after landing or while foraging. The lack of recaptures during the migration period on days after first capture (data from 20 years, exceptions are bad weather days preventing resumption of migration) indicates that the robins stayed on this Alpine pass only for the day of landing. Also there are no breeding birds in this area.

A total of 101 European robins were caught, 62 at night (44 first-year birds, 16 adults, 2 undetermined) and 39 during daylight or at dawn (29 first-years, 9 adults, 1 undetermined). There was no difference in migration phenology between first-year and adult European Robins (nonparametric Mann-Whitney U-Test, P = 0.383, N = 98).

### Blood sampling and body energy stores

The mist nets were continuously surveyed. Blood was collected within a maximum of 10 min (mean 4.32±1.86) after the bird flew into the mist net (determined with a stop-watch) by puncturing the alar vein. Blood samples were centrifuged within 1 h at 3000 rpm for 5 min, and red blood cells were stored in liquid nitrogen for at most 2 weeks and then frozen at −20°C until analysis at the University of Glasgow. Each bird was aged (born in the year of capture or adult; [17]), weighted to the nearest 0.1 g using an electronic balance, and the length of the third outermost primary feather was measured to the nearest 0.5 mm [18]. In European robins, the sex cannot be determined reliably from external characters and thus was not considered. The visible amount of subcutaneous fat deposits between the furcula and on the abdomen was scored on a scale ranging from 0 to 8 [19], with 0 representing no visible fat. These scores correlate well with the amount of fat extracted from whole birds [19]. European robins on autumn migration in Switzerland have low to medium amounts of fat. Their fat scores ranged between 0 and 4 (score 0: N = 3; 1: N = 51; 2: N = 28; 3: N = 12; 4: N = 3; missing: N = 4). The muscle score estimates the thickness of the breast muscle, representing breast muscle protein mass, and ranges from 0 to 3 [20] (score 1: N = 23; 2: N = 65; 3: N = 10; missing: N = 3).

### Evaluation of blood oxidative state

Parameters of oxidative state were measured in red blood cells. We measured concentration of protein carbonyls (PCs), which is a well-established biomarker of oxidative damage to proteins. Carbonyl groups (C = O) are introduced into proteins from free radicals or via reactions with lipid peroxidation products (malondialdehyde and hydroxynonenal); protein carbonylation is mostly irreversible [12]. The blood concentration of PCs increases after muscle-damaging physical effort [9]. In addition to PCs, we measured the activity of glutathione peroxidase (GPx), an antioxidant enzyme that is up-regulated when generation of hydrogen peroxide and, in particular, of hydroperoxides (early oxidative damage derivatives) increases.

The method from Levine et al. ([21]; see also [22]–[23]) was used to quantify the concentration of PCs in red blood cells. All samples were first diluted with distilled water in order to have a concentration of 1 mg protein per ml, as measured by the Bradford protein assay (Bio-Rad Laboratories, Hercules, USA) using bovine albumin as a reference standard. Nucleic acids were removed by adding 1 volume of a 10% solution of streptomycin sulfonate to 9 volumes of sample. Protein carbonyls were derivatized to 2,4-dinitrophenylhydrazone by reaction with 2,4-dinitrophenylhydrazine (DNPH) according to Levine et al. ([21]; see also [22]–[23]). The pellet was precipitated with cold trichloroacetic acid at 20% and then washed three times with a solution 1∶1 of cold ethanol-ethyl acetate. The pellet was finally re-suspended in 350 µl of guanidine hydrochloride 6 M. The absorbance was read at 370 nm. The mean absorbance of the control tubes was subtracted from the mean absorbance of the sample tubes and the extinction coefficient for DNPH (0.022/ µM/cm) was used to calculate the protein carbonyl concentration, which was expressed as nmol mg^−1^ protein.

The Ransel assay (RANDOX Laboratories, Crumlin, UK) was used to quantify the concentration of GPx in haemolysate (red blood cells diluted 1∶40 with diluting agent provided with the assay). This assay is based on the original method of Paglia and Valentine [24] and analyses were carried out according to previous studies (e.g. [23]). The kinetic reaction was followed for 3 min by reading at 340 nm. A blank reaction was subtracted from the sample absorbance. Values were expressed as Units l^−1^ of haemolysate.

GPx and PCs were analysed in 95 samples. Depending on the analysis fewer samples were available because of missing values for fat and/or muscles score. No sample was excluded.

### Statistical analysis

For each bird, time of capture relative to dawn (civil twilight) was calculated, with negative values (i.e. before dawn) indicating captures at night and positive values indicating captures after dawn during the day (thereafter called time since dawn). Time of dawn of each day for the catching site was taken from www.home.datacomm.ch/juergmueller/sonnenaufgang.htm.

General linear models were used to analyse the effect of time since dawn (linear, squared and cubic), age (two classes), fat score (5 classes) and muscle score (3 classes) on either the concentration of GPx or PCs as the dependent variable. Non-significant terms were removed from the final model. The time span between capture and blood sampling (up to 10 min) had no noticeable effect on GPx or PCs, confirming similar findings by [15].

## Results

**Figure 1:**
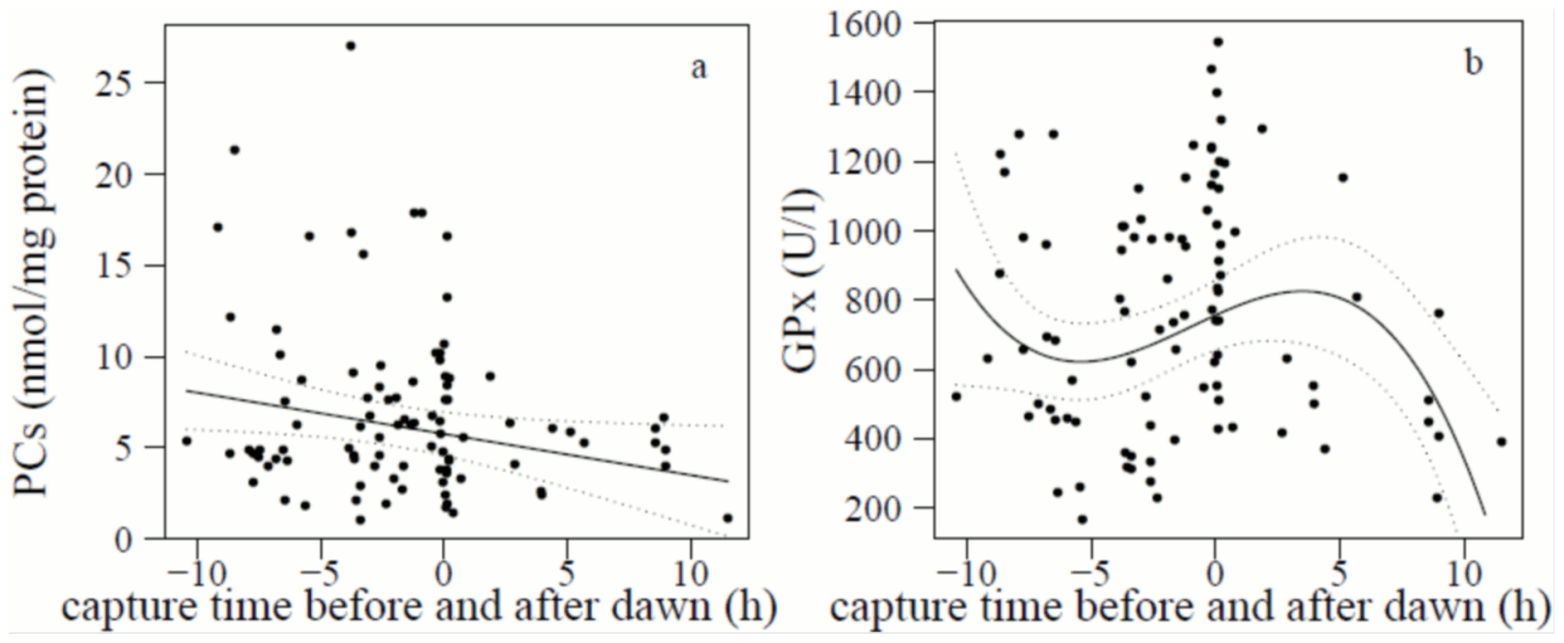
Relationship between (*a*) protein carbonyls (PCs) (nmol/mg protein) of European robins and capture time since dawn, and (*b*) glutathione peroxidase (GPx) (U/l haemolysate) and capture time since dawn (N = 95). Dawn is set to zero. Negative values (i.e. before dawn) indicate captures at night and positive values captures after dawn during the day. Dots are raw data points. The linear and cubic relationships with 95% confidence interval are derived from the models presented in Table 1 and given for muscle score 2 (PCs) and for young birds with muscle score 2 (GPx), respectively.

**Figure 2:**
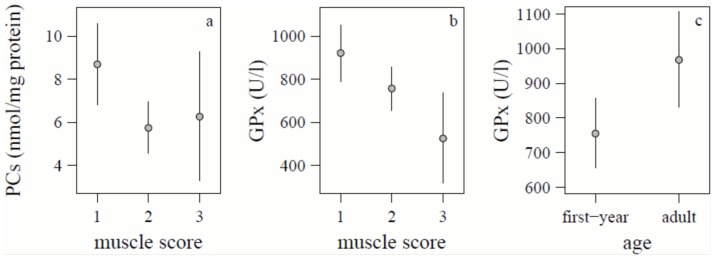
(*a*) Mean (95% confidence intervals) protein carbonyls (PCs) (nmol/mg protein) corrected for the linear relationship with time since dawn for muscle score 1 (N = 23), 2 (N = 63) and 3 (N = 9). For statistics see Table 1. (*b*) Mean (95% confidence intervals) glutathione peroxidase (GPx) (U/l haemolysate) corrected for the cubic relationship with time since dawn and age for muscle score 1 (N = 22), 2 (N = 64) and 3 (N = 9). (*c*) Mean (95% confidence intervals) glutathione peroxidase (U/l haemolysate) corrected for the cubic relationship with time since dawn and muscle for first-year (N = 70) and adult (N = 25) European robins. For statistics see Table 1.

The concentration of PCs was linearly dependent on time since dawn and muscle score, while the quadratic and cubic term of time since dawn and fat score had no significant effect (Table 1, Figure 1a). Also age had clearly no significant effect (P = 0.89) on PCs. PCs in European robins during their nocturnal migratory flight were on average higher and showed a higher variance than during the day when resting and feeding. Robins with a muscle score of 1 showed higher PC levels than those with muscle scores 2 and 3 (Figure 2a).

**Table 1:** Dependence of protein carbonyl (PCs) and glutathione peroxidase (GPx) concentration in European robins on time since dawn (third-order polynomial), muscle score and age in a General linear model.

	Protein carbonyl (nmol/mg protein)				GPx (U/l)			
	Effect ± SE	df	F value	P value	Effect ± SE	df	F value	P value
Time since dawn	−0.23±0.11	1	4.64	0.034	32.71±15.65	1	4.37	0.039
Time since dawn squared				ns	−1.67±1.12	1	1.09	0.132
Time since dawn cubic				ns	−0.58±0.22	1	7.05	0.009
Muscle score		2	3.64	0.030		2	5.57	0.005
score 1	2.42±1.77				392.70±120.40			
score 2	−0.54±1.61				228.58±106.61			
Age (1y)				ns	−212.57±69.38	1	9.39	0.003

N = 95 for both models. Fat score was not significant (ns) and removed from both models; time since dawn cubic and squared and age were not significant in the model for PCs and removed. The effect for the variable muscle score is given for muscle scores 1 and 2 *versus* muscle score 3, and for first-year birds *versus* adults.

The concentration of GPx in red blood cells was significantly dependent on time since dawn in a curvilinear way (third-order polynomial) and on muscle score and age, while fat score had no significant effect (Table 1). GPx concentration of actively migrating European robins were on average high during the first part of the night, slightly lower in the middle part and peaked at dawn. After dawn GPx concentration decreased throughout the day in robins resting and feeding (Figure 1b). Independent of time since dawn and age, the highest GPx concentrations were found in lean birds (muscle score 1), medium levels for muscle score 2 and the lowest levels for muscle score 3 (Figure 2b). Independent of time since dawn and muscle score, adults had significantly higher GPx concentrations than first-year European robins (Figure 2c).

The concentrations of GPx and PCs were significantly correlated (r = 0.303, P = 0.003, N = 95).

## Discussion

This study is, to our knowledge, the first to measure a marker of oxidative damage and of antioxidant capacity in birds caught out of migratory endurance flight. We found higher concentrations of PCs and GPx during the night in flying birds than in resting birds. Both markers also depended on muscle score. PC concentration was high for muscle score 1 only, while GPx concentration increased gradually from the highest to the lowest muscle score. Independently of time of day and muscle score, adults had higher GPx than first-year birds.

Nocturnal migrants trapped at night on the Alpine pass are caught out of their active flight. Radar studies performed at the study site and elsewhere demonstrate that nocturnal migrants appear aloft suddenly after sunset and numbers decrease gradually during night [25]–[26], indicating that most start migration 1–2 h after dusk. However, some migrants may start later during the night (e.g. [27]). Landing may occur throughout the night, but at the latest at dawn. That birds caught at night are in endurance flight is confirmed by measurements of plasma fat and protein metabolites and corticosterone. European robins caught at the study site revealed the typical pattern of an active fasting state in the night captures, which was clearly different from the metabolic patterns of a fasting inactive and a resorptive state. They showed significantly increased fat and protein catabolites and moderately increased corticosterone, typical for unstressed birds during a high physical performance [28]–[32].

Therefore, we conclude that the high PCs and GPx during the night are the consequence of the high metabolic rate during flight, while the low PC and GPx concentrations during the day is the consequence of a reduced metabolic rate while resting and feeding. The high variation in PC and GPx values during the night and the somewhat lower GPx values in the middle of the night might be the consequence of different flight durations caused by European robins starting at different times during the night. The high variation in PC values at dawn may be caused by birds landing at that moment and some having already landed before but starting to move around at dawn. Similar patterns may apply to GPx. A common pattern in both markers however is the absence of high values during the day when robins are definitely not in endurance flight. In order to more completely interpret these patterns, we would need to know more about flight times of the individual robins and of the reaction time of PCs and GPx to exercise and rest.

One might argue that the difference in PC and GPx concentration between day and night could be the result of a diurnal pattern of the two markers. Studies on chicken and rat brain found that GPx has a peak towards the end of the night, a few hours after the peak of melatonin, while it is quite stable over the rest of the day [33]–[34]. In contrast, GPx activity in blood of rats tended to be higher over the daylight hours [35]. The decrease in GPx and PC activity from night to day cannot therefore be ascribed to these diurnal rhythms.

Starvation was shown to have a small impact on daily changes in GPx. Although fasting for 2 days increased GPx activity in chickens [36], a 18-hours and a two-week fast did not cause any effect on GPx activity in rats [37] and in free-living king penguins [38]. The European robins caught at night were certainly in a fasting state; however, since they rest and feed during the day they are not in a starvation phase. Therefore it does not seem likely that GPx activity is affected by starvation.

The significant positive correlation between the two markers indicates that the antioxidant GPx is produced concomitantly with the increase in PCs. The up-regulation of GPx seems to be an adaptation of migrant species to endurance flight to counter the production of pro-oxidants raised by exercise and to maintain redox homeostasis. This is in contrast to the non-migrating homing pigeons. Their non-enzymatic serum antioxidant capacity decreased, and their oxidative damage increased after a 3 h flight, thus their redox homeostasis shifted towards more oxidative conditions [14]. In contrast to free-flying migrants, the flights of homing pigeons are imposed by the experimenter and thus largely involuntary. This agrees with findings in mammals suggesting that acute and unaccustomed exercise often causes an imbalance between an increase in reactive oxygen species (ROS) production and a decrease in antioxidant defence, thus causes oxidative stress, while regular exercise and training up-regulates antioxidant defences to match a constrained ROS production (e.g. [11], [39]–[41]; reviewed in [42]).

Independently of time of day, robins with the lowest muscle score had the highest PC concentration. The fact that fat score did not correlate with PCs indicates that PCs depended on a feature of the flight muscle, rather than on lipid energy stores. We can think of two possible explanations. Firstly, small flight muscles have been found to have a lower efficiency with which metabolic power input is converted into flight mechanical output [43], thus more energy, with possibly a higher ROS and PC production, is used for flight. Secondly, birds with reduced flight muscles usually also have smaller fat stores (as also found in this study) and, consequently, use proportionally more protein and less lipids as fuel than fat birds [2]. If the more complex breakdown of protein and excretion of nitrogen entail the production of more PCs than the metabolically simpler fat oxidation, a proportionally higher protein catabolism would result in a higher concentration of PCs. However, we did not find supporting evidence for this. Also, it is unknown for how long PCs persist after the end of an endurance flight, which might explain the higher PC concentration in resting birds with a low muscle score compared with a high muscle score, a pattern also found in migrating passerines after a sea crossing [13]. It appears that European robins with low muscle scores suffer higher oxidative stress than European robins with high muscle scores and they up-regulate the concentration of GPx concomitantly with the increase in PCs.

Adult European robins had a higher GPx concentration than European robins migrating for the first time (first-year birds) independently of time of day and muscle score, while there was no significant difference in PC concentration. The higher production of GPx could be due to the maturation of the antioxidant machinery, which has been described in many species [44]–[46]. However, in homing pigeons a stronger decrease in non-enzymatic serum antioxidant capacity has been found in older individuals (age range 1–5 years) [14]. Another explanation could be that older birds were better able to up-regulate GPx since they have already experienced migration. Stressful experiences may improve the individual capacity to withstand future episodes of stress (hormesis) [47]. Hence, hormetic priming to migration effort could have caused irreversible phenotypic changes that made adult European robins more resistant (e.g., higher ability to up-regulate GPx when needed) than first-year birds. Finally, it might be possible that a selective disappearance of birds with a low antioxidant protection has taken place.

In summary, the results of this study demonstrate for the first time that a free-flying migrating passerine during endurance flight is indeed exposed to a higher oxidative stress (expressed as protein carbonyl concentration). Antioxidant capacity (expressed as GPx concentration) is up-regulated concomitantly suggesting that free-flying migrants adapt their antioxidant system to the extraordinary endurance exercise of sustained flight at a very high metabolic rate. Due to logistic constraints on the Alpine pass and the small size of the bird, we were restricted to the measurements of only PCs and GPx. It will be very interesting to see whether the concentrations of reactive species and of other markers of oxidative damage are also increased during endurance flight and whether other markers of the antioxidant capacity are similarly up-regulated. This would also allow teasing apart the routes by which the oxidative balance is maintained during endurance flight.

Our data clearly show that oxidative stress may be a further physiological challenge imposed upon by migration. Oxidative stress, therefore, is a factor potentially affecting bird migration strategies which has not yet been taken into consideration. We can see at least two ways in which oxidative stress may shape bird migration. Firstly, migrants may favour a strategy with short non-stop flights and frequent stopovers to periodically decrease pro-oxidants raised by endurance flight to “normal” levels. It is well known that nocturnal passerine migrants migrate at night, but stop over during the day even when crossing deserts where they cannot forage and replenish their energy reserves [26]. Diurnal migrants usually migrate in even shorter hops. It will be interesting to investigate the oxidative balance in species with different migration strategies. The European robin is a short-distance migrant which usually migrates in hops of a night's flight at most over distances of only a few thousand kilometres at maximum. Long-distance migrants which are forced to cross oceans non-stop or the Sahara desert in several consecutive bouts of entire night's flights, or waders with non-stop flights of up to 9 days [3] may show different patterns of RONS production, antioxidant capacity and resulting oxidative stress than European robins. Secondly, it appears that migrants should avoid the breakdown of their flight muscles beyond a certain degree (muscle score 2 in the European robin). It is well known that flight muscles are catabolized concomitantly with decreasing body mass (due to the catabolism of lipids as major fuel), thereby matching flight capacity to decreasing body mass [48]. A further reduction in flight muscle mass, however, should be avoided, since it may not only increase the risk of starvation, but also increase oxidative stress, as suggested by our data. Such a further catabolism of flight muscles occurs when lipid stores are nearing depletion and an increase in corticosterone triggers an increase in protein breakdown [28], or when dehydration brings about a shift in fuel types in favour of more energy derived from protein, resulting in a greater gain of water than from lipids [1], [49]. The risk to catabolize flight muscles too much increases with the duration of non-stop flights and with the risk of not finding adequate stopover habitats, a situation occurring in inhospitable areas or when displaced by unfavourable wind. This in turn would be an argument to carefully select favourable weather conditions and to avoid long non-stop flights when predictions about weather conditions are more difficult.

It is increasingly recognised that balancing investment in antioxidant defences against the consequences of sustaining oxidative damage has possibly influenced life history traits [50], [42]. An open question in this context is what costs are incurred by antioxidant production and an up-regulation of the antioxidant capacity, and hence whether maintaining and up-regulating antioxidant capacity may compromise other physiological processes. This is key to a deeper insight of the possible effects of oxidative stress on migration strategies.
